# Quaternary prevention: reviewing the concept: Quaternary prevention aims to protect patients from medical harm

**DOI:** 10.1080/13814788.2017.1422177

**Published:** 2018-01-31

**Authors:** Carlos Martins, Maciek Godycki-Cwirko, Bruno Heleno, John Brodersen

**Affiliations:** ^a^ Family Medicine, Department of Community Medicine, Information and Decision in Health (MEDCIDS) of the Faculty of Medicine of Porto, Centre for Health Technology and Services Research (CINTESIS) Porto Portugal; ^b^ Division of Public Health, Centre for Family and Community Medicine, Medical University of Lodz Lodz Poland; ^c^ Chronic Diseases Research Centre, NOVA Medical School/Faculdade de Ciências Médicas, Universidade NOVA de Lisboa Lisboa Portugal; ^d^ Section of General Practice & Research Unit for General Practice, Department of Public Health, Faculty of Health Sciences, Primary Healthcare Research Unit, University of Copenhagen Region Zealand Denmark

**Keywords:** Prevention, quaternary prevention, overdiagnosis, patient involvement (empowerment, self-management), health ethics

## Abstract

**Background:** According to the Wonca International Dictionary for General/Family Practice Quaternary Prevention is defined as: ‘Action taken to identify patient at risk of overmedicalization, to protect him from new medical invasion, and to suggest to him interventions, which are ethically acceptable.’ The concept of quaternary prevention was initially proposed by Marc Jamoulle and the targets were mainly patients with illness but without a disease.

**Objectives:** The purpose of this opinion article is to open the debate around a new possible definition and a new conceptual model of quaternary prevention based on the belief that quaternary prevention should be present in physicians’ minds for every intervention they suggest to a patient.

**Discussion:** The debate around quaternary prevention is vital in the context of contemporary medicine and has expanded worldwide. The human being may suffer harm from medical interventions from conception, during their childhood, during their entire healthy lifetime as well as during a self-limited disease, a chronic disease, or a terminal disease. The current definition of quaternary prevention has limitations because it excludes patients and medical interventions where a quaternary prevention perspective would be needed and useful to protect patients from harm. In this context, a new definition and conceptual model of quaternary prevention is proposed.

**Conclusion:** In this new proposal, quaternary prevention is defined as an ‘action taken to protect individuals (persons/patients) from medical interventions that are likely to cause more harm than good.’

KEY MESSAGES

- Current definition of quaternary prevention has limitations.
- Quaternary prevention aims to protect people and patients from medical harm.
- Quaternary prevention is an inevitable concept in good clinical practice.

## Introduction

The current definition of quaternary prevention may have some limitations because it excludes patients and medical interventions where a quaternary prevention perspective would be needed and useful to protect patients from harm. The purpose of this opinion article is to open the debate around a new possible definition and a new conceptual model of quaternary prevention based on the belief that quaternary prevention should be present in physicians’ minds for every intervention they suggest to a patient.

## There are several forms of prevention

In the last fifty years, three main categories of prevention have been considered: primary, secondary, and tertiary [1,2]. These three separate categories were defined by the World Organization of Family Doctors (Wonca**)** International Dictionary for General/Family Practice in 2003 as: primary prevention—‘action taken to avoid or remove the cause of a health problem in an individual or a population before it arises;’ secondary prevention—‘action taken to detect a health problem at an early stage in an individual or a population, thereby facilitating a cure or reducing or preventing it spreading or long-term effects’ (e.g. screening, case finding and early diagnosis); tertiary prevention—‘action taken to reduce the chronic effects of a health problem in an individual or a population by minimizing the functional impairment consequent to the acute or chronic health problem’ (e.g. prevent complications of diabetes) [3]. Primary prevention includes some health promotion and specific protection (e.g. immunization), and tertiary prevention includes rehabilitation. As described in the definition by Wonca, the three different types of prevention can be person oriented or oriented at the macro level of society.[Boxed-text B2]

Box 1. Quaternary prevention definition.

**Table:** 

**The current Wonca International Dictionary definition**
‘Action taken to identify patient at risk of overmedicalization, to protect him from new medical invasion, and to suggest to him interventions, which are ethically acceptable.’
**The new definition**
‘Action taken to protect individuals (persons/patients) from medical interventions that are likely to cause more harm than good.’

The idea of preventing illness and disease is attractive for patients and physicians. The belief in early detection, some areas of health politics and financial interest have contributed to the popularity of preventive activities and to the medicalization of everyday life. The emphasis on prevention has led to the growing popularity of the periodic health examination, also called a general health check-up. Patients and physicians tend to overestimate the benefits and underestimate the harms of preventive and curative interventions [4,5]. Medical consultations with healthy people consist in an encounter between a physician and a patient without disease or illness, i.e. between a physician and a patient that is well and feels well. When feeling ill, the patient will supposedly benefit from a medical consultation, but if the patient feels well the probability of getting additional benefit from a medical consultation is lower. However, there is also the possibility of being harmed by excessive medical interventions and even overtreatment. Furthermore, there are also patients who may feel ill without having a disease; these patients are also at an increased risk of being harmed by overtesting and overmedicalization. The need to reduce these risks in the latter case, where the patient has an illness with no disease, was raised by Marc Jamoulle in 1986 with a new category of medical prevention: the quaternary prevention [6].

## The concept of quaternary prevention

In this proposal, Jamoulle made an important contribution to the academic and scientific debate about the role of prevention and possible harm of medical activities implemented with preventive intention. In 1999, the quaternary prevention concept was also integrated in the Wonca International Dictionary for General/Family Practice and was defined as: ‘Action taken to identify patient at risk of overmedicalization, to protect him from new medical invasion, and to suggest to him interventions, which are ethically acceptable’ [3].

**Figure 1.**
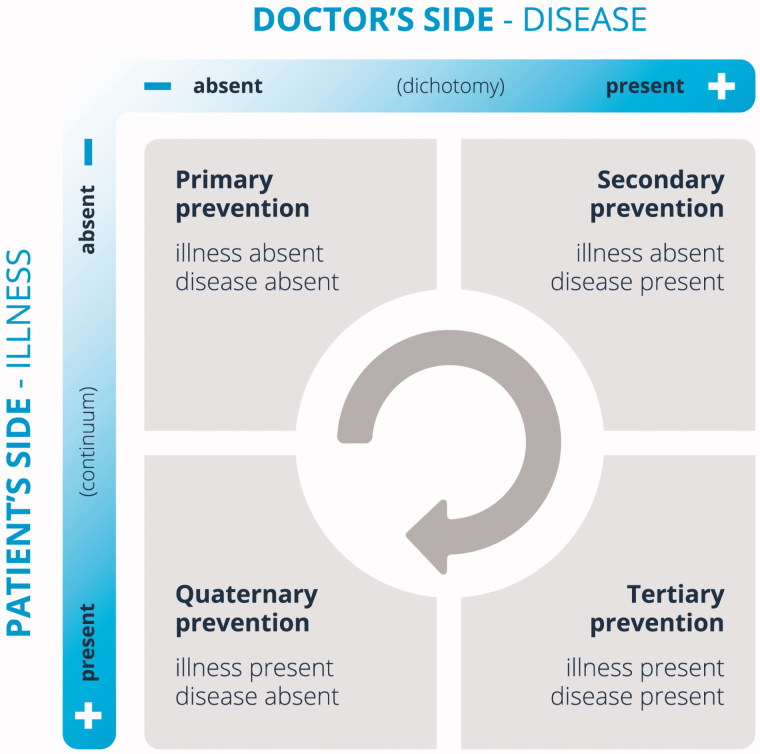
Model of different categories of prevention in the relational model proposed by Marc Jamoulle. Adapted from the original with permission [7].

In elaborating his proposal, Jamoulle also presented a model of interpretation of the four categories of prevention (Figure 1) [7]. It hinges on the conceptual distinction between illness, a subjective experience of poor health, and disease, a theoretical construct based on pathophysiology. These two concepts can be used to map four areas based on a combination of illness and disease status (Figure 1). In Jamoulle’s elaboration of the model, the field of action of quaternary prevention would be the only situation in which the patient would have illness without having disease. The typical example would be a patient with bio-medically or psychiatrically unexplained symptoms. Quaternary prevention would mean that the physician should refrain from doing potentially harmful invasive testing in such patients.

This fact is a limitation of Jamoulle’s model, and it may contradict the previously presented quaternary prevention definition. People in the three remaining quadrants of Figure 1 are also at risk of overmedicalization, e.g. overuse, overtreatment, and overdiagnosis, and also need protection from unnecessary and ethically questionable medical examinations and interventions.

## Harm associated with preventive medical interventions

The debate around quaternary prevention is vital in the context of contemporary medicine. It should be present in the mind of every healthcare professional when they suggest an intervention to one of their patients.

In the primary prevention field, some preventive interventions have important health benefits (e.g. polio immunization). However, there may be also some interventions that pose significant harm. For example, the influenza immunization campaign during the recent influenza pandemic produced significant harm in hundreds of children who now suffer from narcolepsy caused by the vaccine [8].

Consider general health checks as an example of secondary prevention. They do not reduce morbidity or mortality, they do not reduce overall risk of cardiovascular or cancer-related disease, and they increase the number of new diagnoses [9]. However, a high proportion of the occidental population think they should undergo routine medical tests with a clear tendency towards overuse of different tests [10,11]. This pattern of behaviour constitutes a modern health risk factor. Getting false-positive diagnoses, finding incidentalomas, being overdiagnosed, and being exposed to a cascade of follow-up procedures are some of the harms that may significantly impair the quality of life of healthy people undergoing health checks or other forms of medical screenings [12–15].

There are plenty of examples of harm related to tertiary prevention. The classical example is the use of antiarrhythmic drugs in post myocardial infarction that reduced arrhythmias but increased mortality [16]. Another well-known example is the use of hormone replacement therapy that not only failed to reduce cardiovascular mortality, but increased the number of cases of breast cancers, stroke, and thromboembolic events [17]. More recently intensive glycaemic control was shown to reduce the average HbA1c but did not reduce mortality [18,19]. These are all good examples of well-intentioned tertiary prevention that were already in place before solid randomized controlled trial evidence was available. Quaternary prevention also involves refraining from providing therapy that has not been adequately assessed in a randomized controlled trial with low risk of bias.

## Harm associated with medical interventions beyond the preventive interventions

Many factors contribute to a more intensive exposure to medicine. Often this has positive aspects, but it also has negative aspects. Disease mongering campaigns, widening a disease definition, and lowering the normal thresholds related to some chronic diseases are some of the mechanisms that turn healthy persons into patients.

Disease mongering campaigns frequently originate from and are guided by economic motivations. They may create insecurity in healthy persons and cause them to seek unnecessary medical care. In the end, this may lead to overuse, overtreatment, and overdiagnosis [20].

Lowering the normal thresholds verified in some highly prevalent pathologies, for example diabetes and hypertension, also suddenly transform thousands of healthy persons into ‘pathology labelled’ patients. This also contributes to the growth of multimorbidity, polypharmacy, overtreatment, and a greater exposition to the medication’s side effects and harms [12].

All these factors contribute to a Medicine that is more interventive and more invasive than ever but, unfortunately, also with greater chances of harm. And this makes quaternary prevention more needed than ever.

## Quaternary prevention: the need of a new definition

A new definition should clearly state that people in all quadrants of Figure 1 may be eligible for quaternary prevention.

This is why we propose a revision to Wonca’s definition of quaternary prevention, and strongly support the definition of quaternary prevention proposed by Brodersen et al., that defines quaternary prevention as an action taken to protect individuals (persons/patients) from medical interventions that are likely to cause more harm than good [21].

**Figure 2.**
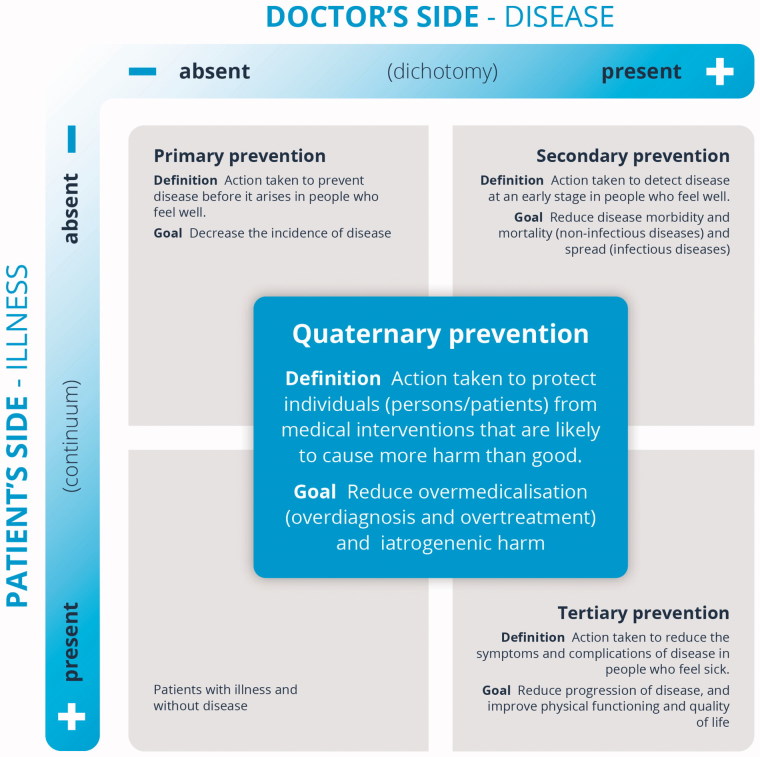
Illness and disease in relation to the four categories of prevention. Adapted from the original with permission [21].

With this new definition, Brodersen et al. also propose a new conceptual model (Figure 2). In this new conceptual model, the quaternary prevention field expands and moves into the centre of the axes of illness and disease. This does not mean that the previous quadrant (illness/no disease) where Jamoulle allocated the quaternary prevention is now empty. Patients in that quadrant are patients with illness and without disease. Frequently, labels like ‘medically unexplained symptoms,’ ‘functional disorders’ or ‘bodily distress syndrome’ are associated with patients in this quadrant. However, all these labels have limitations and may stigmatize these patients even further. Hopefully, many of these patients will benefit from future research conducted in a perspective of medical scientific theory but also in perspectives from other science theories, e.g. social science, anthropology, etc. We agree that these patients are indeed at risk of overtreatment and harm. This continues to be a quaternary prevention field, but not the only one. The other three quadrants will also be fields of quaternary prevention because in the other quadrants there are also citizens and patients at risk of overmedicalization, overtreatment and harm. This model and the definition clarify that quaternary prevention should be present in physicians’ minds for every intervention they suggest to a patient. In contemporary medicine, the human being may suffer harm from medical interventions from conception, during their childhood, during their entire healthy lifetime as well as during a self-limited disease, a chronic disease, or a terminal disease. The quaternary prevention concept aims to make this reality recognized by health professionals and patients. It goes beyond preventing overdiagnosis or preventing overtreatment; it includes preventing all types of harm associated with medical interventions.

In Jamoulle’s conceptual model, the central arrow moving from primary prevention towards quaternary prevention suggests the idea of a natural sequence of the different levels of prevention (Figure 1). The association between the natural history of the disease and the proposal of the three classical steps of prevention may also be related with this arrow. However, we think that this sequence is out of touch with current public health and general practice and even more so when we think about quaternary prevention. The ‘real’ patients may be in one or all of the quadrants; whichever quadrant these patients may be in, they might benefit from quaternary prevention.

Furthermore, this new definition is more in line with the current academic thinking about quaternary prevention. Since Jamoulle’s initial proposal, there has been growing academic debate about this concept that has expanded worldwide [15,22–24]. In more recent publications, Marc Jamoulle himself states that quaternary prevention affects the other three levels of preventive activities [25–27]. Another relevant point is the growing consensus among different authors about the close relation among the concept of quaternary prevention and the non-maleficence principle of medical ethics usually mentioned as *primum non nocere* (first, do no harm) [15,25,28–30]. Wagner H states ‘The concept of quaternary prevention is nothing more than the systematization of the concept of *“primum non nocere”* in our modern medical practice, an ethical approach to practice better clinical care and to protect people of excess of medicine’ [30]. In this new definition, the focus on avoiding harm to patients is more perceptible. Another aspect that favours this new definition is the language that is used. This new definition is simpler and easier to understand, both for patients and health professionals. In a world where health literacy is still a frequent problem, this is not a minor issue.

Finally, we would like to state that quaternary prevention should not be faced as a panacea, nor should it be faced as a risk-free medical activity. With the intention of minimizing harms, there is the risk of refusal of some medical interventions that would indeed benefit some patients. However, this is always an inherent characteristic of the uncertainty of medicine, especially general practice. And under this perspective, the new definition is also more reasonable as it incorporates the notion of likelihood.

## Conclusion

We think that the current definition and the conceptual model of quaternary prevention have some limitations and, therefore, we present a new definition and an alternative model that can contribute to facilitate dissemination and future research related to this relevant concept of contemporary medicine.
